# Clec7a-targeted Res@GelMA hydrogels regulate macrophage polarization to reduce neuroinflammation and promote spinal cord repair

**DOI:** 10.1186/s13018-025-06631-0

**Published:** 2026-01-24

**Authors:** Zhonglian Zhu, Jiankang Chang, Xubin Gao, Zhaodong Wang, Keyou Duan, Jianzhong Guan

**Affiliations:** 1 Department of Orthopedics, The First Affiliated Hospital of Bengbu Medical University, No. 287, Changhuai Road, Bengbu City, 233030 Anhui Province China; 2 Anhui Province Key Laboratory of Tissue Transplantation, Bengbu Medical University, Bengbu, 233030 Anhui China; 3 Graduate School, Bengbu Medical University, Bengbu, 233030 Anhui China

**Keywords:** Spinal cord injury, Macrophage polarization, Clec7a, Res@GelMA, Neuroinflammation

## Abstract

Excessive inflammation driven by macrophage phenotype imbalance is a key pathological barrier hindering neural repair after spinal cord injury (SCI). Here, methacryloyl gelatin hydrogel (GelMA) loaded with natural anti-inflammatory agent resveratrol (Res) was designed and synthesized. Scanning electron microscopy (SEM), x-ray diffraction (XRD), fourier-transform infrared spectroscopy (FT-IR), Ultraviolet (UV) and rheological characterization confirmed that Res-loaded GelMA hydrogel (Res@GelMA) was successfully synthesized. High-Performance Liquid Chromatography (HPLC) analysis demonstrated sustained Res release. CCK8 and cell adhesion experiments confirmed that Res@GelMA treatment did not affect the cell function of RAW264.7 and had good cell compatibility. Flow cytometry, Enzyme-Linked Immunosorbent Assay (ELISA) and western blot assays revealed that Res@GelMA treatment promoted RAW264.7 to M2 polarization, while reducing the levels of inflammatory factors (IL-1β, IL-6, TNF-α) and down-regulating the expression of IL-1R1/MyD88/TNFR1 inflammatory signaling proteins. Transcriptome sequencing combined with functional screening identified C-type lectin receptor Clec7a as a key target gene regulated by Res@GelMA. Importantly, knockdown of Clec7a and Res@GelMA were both anti-inflammatory, promoted M2 polarization, and blocked the activation of the TLR2/TLR4-p38 MAPK signaling axis. In the SCI mouse model, local implantation of Res@GelMA significantly improved tissue pathological damage and enhanced motor function recovery compared with free Res or blank GelMA. In addition, Res@GelMA achieved systemic anti-inflammation by downregulating the Clec7a-TLR-p38 pathway in the injured area and promoting M2 polarization. This study developed an anti-inflammatory hydrogel material that can regulate the phenotype of macrophages, laying a theoretical and technical foundation for the development of neural repair strategies targeting the inflammatory microenvironment.

**Supplementary Information:**

The online version contains supplementary material available at 10.1186/s13018-025-06631-0.

## Introduction

SCI is a severe trauma to the central nervous system that not only causes direct damage to neurons and axons but also triggers a complex cascade of secondary injury reactions [1, 2]. SCI has posed a significant public health burden, with an estimated annual incidence of 10.4–83 cases per million people worldwide [3], and approximately 37.5 cases per million people in China [4]. This leads to significant psychological and economic burdens for patients and their families [5, 6]. To date, treatments such as medications [7], mitochondrial transplantation [8], and stem cell transplantation [9] have been less than ideal and have associated toxic side effects. Therefore, it is necessary to continue exploring new, more effective and safer approaches for clinical treatment.

Excessive and persistent neuroinflammatory responses, particularly abnormal macrophage activation and polarization imbalance, are widely considered to be the core pathological factors hindering neuroregeneration and functional recovery in SCI patients [10–14]. Macrophages exhibit two classical polarization states: the pro-inflammatory M1 phenotype and the anti-inflammatory/reparative M2 phenotype [15]. Research has shown that when the M1 phenotype predominates within the injury microenvironment, it can trigger a robust inflammatory response [16]. These further releases large amounts of pro-inflammatory mediators, reactive oxygen species, and various neurotoxic substances [17]. This exacerbates tissue damage, promotes neuronal apoptosis and axonal rupture, and ultimately forms glial scars that are detrimental to axonal regeneration [18, 19]. Therefore, exploring how to effectively induce macrophages in the injured area to switch from the M1 phenotype to the M2 phenotype, which is more conducive to neuroregeneration, and thus reshape the inflammatory microenvironment, is a promising research direction [20].

Currently, high-dose methylprednisolone sodium succinate (MPSS) remains one of the limited pharmacological options applied in the acute phase of SCI, primarily for its anti-inflammatory and neuroprotective effects. However, the clinical use of MPSS is highly controversial due to its well-documented and severe side effects, including increased risks of gastrointestinal bleeding, infection, hyperglycemia, and avascular necrosis, with only marginal and debated long-term benefits [21, 22]. Moreover, the broad immunosuppressive action of MPSS lacks specificity in modulating the nuanced balance of macrophage polarization, often leading to non-selective immunosuppression. In contrast, resveratrol (Res), as a natural compound, has demonstrated a superior safety profile. It has demonstrated therapeutic potential in various disease models due to its remarkable anti-inflammatory, antioxidant, neuroprotective, and immunomodulatory activities [23]. Studies have shown that resveratrol can effectively inhibit the polarization of macrophages to the M1 phenotype in the context of neuroinflammation by regulating SIRT1, Nrf2/HO-1, and inhibiting NF-κB, while enhancing their polarization to the M2 phenotype, thereby alleviating inflammatory responses and tissue damage [24, 25]. However, resveratrol’s poor water solubility, rapid metabolism, low bioavailability, and inadequate targeting to damaged areas severely hinder its clinical translation [26]. Achieving effective, sustained, and safe delivery to the injured area is a key challenge in unleashing its therapeutic benefits.

Methacrylamide-modified gelatin (GelMA) hydrogels have become a research hotspot in tissue engineering and drug delivery systems (DDS) due to their excellent biocompatibility, biodegradability, tunable physical and chemical properties (such as hardness, pore size, and degradation rate), and similarity to the native extracellular matrix (ECM) [27]. Their photocurability is particularly suitable for in situ injection applications and can provide a physical barrier to protect vulnerable neural structures [28]. Using GelMA as a sustained-release carrier for resveratrol has the potential to overcome its pharmacokinetic drawbacks, ensuring sustained, localized release of the drug at the core of the injury, maximizing its therapeutic efficacy by modulating the immune microenvironment. We therefore hypothesized that local, sustained delivery of resveratrol via GelMA hydrogel (Res@GelMA) would ameliorate neuroinflammation and promote functional recovery after SCI by modulating macrophage polarization through the targeted downregulation of Clec7a.

Therefore, this study aimed to construct resveratrol-loaded GelMA hydrogels (Res@GelMA), screen for differentially expressed gene clusters based on transcriptome sequencing, and identify the core target, Clec7a, through bioinformatics analysis. By using Res@GelMA and intervening with Clec7a, we assessed the polarization state of macrophages at the site of spinal cord injury and their therapeutic potential for SCI. This study not only aims to elucidate the core regulatory role of Res@GelMA in the SCI inflammatory microenvironment through downregulation of Clec7a, but also provides a solid theoretical and experimental basis for the development of effective SCI repair strategies based on immune modulation.

## Materials and methods

### Material synthesis and characterization

Methacrylated gelatin (GelMA) was purchased from Sigma-Aldrich (St. Louis, MO, USA), and Res was purchased from Aladdin (Shanghai, China). The Res-loaded GelMA hydrogel (Res@GelMA) was prepared via physical blending. Briefly, Res (20 mg/mL) was dissolved in a GelMA prepolymer solution (10% w/v in phosphate buffered saline (PBS), pH 7.4) and stirred at room temperature in the dark for 2 h to ensure homogeneity and drug loading. The photoinitiator lithium phenyl-2,4,6-trimethylbenzoylphosphinate (LAP) was then added to the mixture at a final concentration of 0.25% (w/v) and gently stirred until fully dissolved. Photocrosslinking was performed under a 365 nm UV lamp (OmniCure S2000, Lumen Dynamics, Canada) at an intensity of 10 mW/cm² for 60 s. The freshly prepared hydrogels were used immediately for characterization or cell experiments. For short-term storage (up to 24 h), hydrogels were sealed and kept in PBS at 4 °C in the dark to maintain stability. The hydrogel morphology was observed using scanning electron microscopy (SEM, Hitachi SU8010, Japan) after samples were frozen in liquid nitrogen, lyophilized, and sputter-coated with gold. XRD analysis was performed using a Bruker D8 Advance diffractometer (Billerica, MA, USA) (Cu-Kα radiation, λ = 1.5406 Å, scanning range 5°–50°). FT-IR was conducted on a Thermo Fisher Nicolet iS50 spectrometer (Waltham, MA, USA) (wavenumber range 4000–500 cm⁻¹). Photocrosslinking was performed under a 365 nm UV lamp (OmniCure S2000, Lumen Dynamics, Canada) (intensity 10 mW/cm², time 60 s). Rheological properties were measured using an Anton Paar MCR 302 rheometer (Graz, Austria) (frequency sweep range 0.1–10 Hz, strain 1%). The in vitro release profile of Resveratrol was determined by high-performance liquid chromatography (HPLC, Agilent 1260 Infinity II, Santa Clara, CA, USA) under the following conditions: C18 reversed-phase column (Agilent ZORBAX Eclipse Plus, 4.6 × 250 mm, 5 μm), mobile phase acetonitrile: water (50:50 v/v), flow rate 1.0 mL/min, detection wavelength 306 nm.

### Cell culture and in vitro experiments

The mouse macrophage cell line RAW264.7 was purchased from the Cell Bank of the Chinese Academy of Sciences (Shanghai, China). Cells were cultured in DMEM high-glucose medium (Corning, USA) supplemented with 10% fetal bovine serum (FBS, Gibco, USA) and 1% penicillin/streptomycin (HyClone, USA) at 37 °C under 5% CO_2_.

## CCK-8 assay

Cell viability was assessed using a CCK-8 kit (Beyotime, Shanghai, China). Briefly, RAW264.7 cells were seeded in 96-well plates (1 × 10^4^ cells/well) and cultured overnight. Then, the culture medium was replaced with 100 µL of fresh medium containing the respective treatments: Control (PBS), Res (10 µM), GelMA (10%), or Res@GelMA (containing 10 µM Res). The cells were exposed to these treatments for 24 h. CCK-8 reagent was then added and incubated for 2 h, followed by measurement of absorbance at 450 nm using a microplate reader (BioTek Synergy H1, Winooski, VT, USA).

### Cell adhesion assay

RAW264.7 cells (5 × 10^2^) were seeded into 6-well plates in 2 mL of complete medium per well and allowed to attach overnight. The medium was then replaced with 2 mL of fresh medium containing the following treatments: Control (PBS), GelMA (10%), or Res@GelMA (containing 10 µM Resveratrol in 10% GelMA). The cells were cultured for 7 days with the medium being replaced every two days to maintain the treatment conditions. Cells were fixed with 4% paraformaldehyde for 15 min at room temperature (RT), washed three times with PBS, and stained with 0.1% crystal violet for 0.5 h at RT. After three washes with PBS, 50 µL/well of 0.5% Triton X-100 (diluted in PBS) was added and incubated overnight at RT to dissolve adherent cells. Cell colony formation was monitored and recorded using an inverted microscope. The number of colonies was quantified using ImageJ software (v1.43u, USA).

### Macrophage polarization analysis

Macrophage polarization was analyzed by flow cytometry. Briefly, RAW264.7 cells (at passages 5–15 and with a viability > 95%) were seeded and treated as indicated. After treatment, cells were harvested, washed twice with cold Hank’s Balanced Salt Solution (HBSS) supplemented with 2% fetal bovine serum (FBS), and then resuspended in the same buffer. Cells were incubated with the corresponding fluorescently conjugated antibodies for 30 min at 4 °C in the dark. After staining, cells were washed twice, resuspended in staining buffer, and analyzed immediately on a BD FACSCelesta flow cytometer (San Jose, CA, USA). A minimum of 10,000 events per sample were collected. Data were analyzed using FlowJo software (version 10.8.1, BD Life Sciences). The following antibodies were used: APC-CD86 (Clone GL1, BioLegend, San Diego, CA, USA), PE-iNOS (Clone CXNFT, eBioscience, San Diego, CA, USA), FITC-CD206 (Clone C068C2, BioLegend), PE-Arg-1 (Clone A1exF5, eBioscience).

## Enzyme-linked immunosorbent assay (ELISA)

Cytokines (IL-1β, IL-6, TNF-α, IL-10, TGF-β) in cell supernatants were measured using ELISA kits (Beyotime, China). The specific catalog numbers were: IL-1β (PI301), IL-6 (PI325), TNF-α (PT512), IL-10 (PI522), TGF-β (PT878). All kits were from the same manufacturing lot to ensure consistency. Briefly, cell supernatants were collected by centrifugation. Total protein concentration in each supernatant was measured using the bicinchoninic acid (BCA) method and cytokine concentrations are reported as absolute values in pg/mL. Then, add 100 µL of standard or supernatant to an antibody-coated 96-well plate and incubate at 37 °C for 90 min. After incubation, the liquid was discarded, and each well was washed four times with 1× Wash Buffer. Add 100 µL of biotinylated detection antibody and incubate at 37 °C for 60 min. Wash the plate four more times. Add 100 µL of horseradish peroxidase (HRP)-labeled streptavidin and incubate at 37 °C in the dark for 30 min. After washing the plate, add 90 µL of 3,3′,5,5′-Tetramethylbenzidine (TMB) substrate and develop color in the dark for 15 min. Add 50 µL of stop solution to terminate the reaction. According to the ELISA kit instructions, the optical density (OD) of each well was measured at 450 nm using a microplate reader, and the corresponding factor levels in each group were calculated. Each sample was run in duplicate, and experiments were repeated in three independent biological replicates (*n* = 3).

### Western blot analysis

Cells were lysed using RIPA buffer (Beyotime, China, Cat#P0013B) supplemented with protease/phosphatase inhibitors (Roche, Switzerland, Cat#04906837001). Protein concentration was determined using a BCA assay kit. Protein samples (30 µg per lane) were separated by 10% SDS-PAGE and transferred onto Polyvinylidene fluoride (PVDF) membranes (Millipore, USA, Cat#IPVH00010). Membranes were blocked with 5% (w/v) skim milk for 1 h and incubated overnight at 4 °C with primary antibodies against: IL-1R1 (1:1000, ab106278, Abcam), MyD88 (1:1000, ab2064, Abcam), TNFR1 (1:1000, ab19139, Abcam), Clec7a (1:1000, ab213684, Abcam), TLR2 (1:1000, ab213357, Abcam), TLR4 (1:1000, ab22048, Abcam), p-P38 (1:1000, ab4822, Abcam), P38 (1:1000, ab170099, Abcam), iNOS (1:1000, ab178945, Abcam), CD86 (1:1000, ab239075, Abcam), Arg1 (1:1000, ab91279, Abcam), CD206 (1:1000, ab64693, Abcam), GAPDH (1:1000, ab8245, Abcam). After incubation with primary antibodies, membranes were washed three times for 10 min each with TBST. Then, membranes were incubated with HRP-conjugated goat anti-rabbit or anti-mouse secondary antibodies (Cell Signaling Technology, 1:3000 dilution) for 1 h at room temperature. Following secondary antibody incubation, membranes were washed another three times for 10 min each with TBST. Protein bands were visualized using electrogenerated chemiluminescence (ECL) chemiluminescence reagent (Advansta, USA, Cat#K-12045-D20) and quantified using ImageJ software. The optical density of each target protein band was normalized to that of the GAPDH band from the same sample.

## Transcriptome sequencing and bioinformatics analysis

Total RNA was extracted from control and Res@GelMA-treated RAW264.7 cells (*n* = 3 biologically independent samples per group) using TRIzol reagent (Invitrogen, USA) following the manufacturer’s protocol. Briefly, RNA was isolated through phase separation with chloroform, precipitated with isopropanol, and washed with 75% ethanol. The final RNA pellet was resuspended in RNase-free water. RNA concentration and purity were measured using a NanoDrop spectrophotometer, with all samples exhibiting A260/A280 ratios between 1.8 and 2.1. RNA integrity was verified using an Agilent 2100 Bioanalyzer, and only samples with an RNA Integrity Number (RIN) > 7.5 were used for subsequent library construction. After quality assessment using an Agilent 2100 Bioanalyzer (Santa Clara, CA, USA), mRNA library construction and Illumina NovaSeq 6000 sequencing (150 bp paired-end) were performed by Novogene (Beijing, China). Raw data underwent quality control with FastQC. Reads were aligned to the mouse reference genome (GRCm39, Ensembl release 109) using HISAT2, and gene expression quantification was performed using featureCounts. Low-expression genes were filtered out by requiring a normalized count of > 10 in at least three samples. Differential gene expression analysis was performed using the DESeq2 R package (|log2FC|>1, *p* < 0.05). Gene Ontology (GO) and Kyoto Encyclopedia of Genes and Genomes (KEGG) pathway enrichment analyses were performed using the clusterProfiler R package. Macrophage-related gene sets (“GOBP_MACROPHAGE_DIFFERENTIATION” and “GOBP_MACROPHAGE_ACTIVATION”) were sourced from MSigDB (Broad Institute, Cambridge, MA, USA). The raw RNA-seq data generated in this study are available from the corresponding author upon reasonable request.

## Cell transfection

RAW264.7 cells were seeded into 6-well plates at a density of 5 × 10^6^ cells per well and cultured overnight to reach 60–70% confluence. Cells were transiently transfected with synthetic small interfering RNAs (siRNAs) targeting Clec7a using Lipofectamine 3000 reagent (Invitrogen) according to the manufacturer’s protocol. The siRNA (final concentration: 50 nM) and Lipofectamine 3000 were diluted in Opti-MEM reduced serum medium. The transfection mixture was incubated with the cells for 6 hours, after which it was replaced with fresh complete culture medium. The cells were then cultured for an additional 42 h (total post-transfection time of 48 h) before subsequent experiments. Three different siRNA sequences (siClec7a-1, siClec7a-2, siClec7a-3) were designed and synthesized by GenePharma (Shanghai, China) to ensure targeting specificity and to screen for optimal knockdown efficiency. Transfection efficiency and knockdown efficacy were validated for each siRNA by both qPCR and western blot analysis. Based on this validation, siClec7a-1 demonstrated the highest knockdown efficiency and was therefore selected for all subsequent functional experiments. The siRNA sequences used were:

siClec7a-1: 5′-GCUCAAGAUCAUCGACAAUTT-3′ (sense).

siClec7a-2: 5′-CCACAGAGUUCUCAUCCAATT-3′ (sense).

siClec7a-3: 5′-GGACAUCAAGUACGAGUAUTT-3′ (sense).

Negative control siRNA (siCtrl): 5′-UUCUCCGAACGUGUCACGUTT-3′.

### Quantitative real-time PCR (qPCR)

Total RNA was extracted using TRIzol reagent according to the manufacturer’s protocol (Life Technologies, USA). For each sample, 1 µg of total RNA was reverse-transcribed into cDNA. qPCR was performed using TB Green Premix Ex Taq II (Takara, Japan) on a QuantStudio 6 system (Applied Biosystems, USA). Each 20 µL reaction mixture contained 2 µL of diluted cDNA template. All samples were run in duplicate (technical replicates). The reaction program was: 95 °C for 30 s; 40 cycles of 95 °C for 5 s and 60 °C for 30 s; followed by a melt curve analysis to verify primer specificity and the absence of primer-dimers or non-specific amplification. The amplification efficiency for each primer pair was validated prior to the experiment by generating a standard curve with a series of cDNA dilutions, and all primers used exhibited efficiencies between 90% and 110%. GAPDH was selected as the internal reference gene after validation. We confirmed that the expression of GAPDH remained stable and was not significantly altered across our experimental groups (including Res@GelMA treatment and Clec7a knockdown). Primers synthesized by Sangon Biotech (Shanghai, China) were:

Clec7a-F: 5′-TGCTGGTGGTGTTCAACTTC-3′.

Clec7a-R: 5′-TGGTAGGTGGCATTGTTGAG-3′.

GAPDH-F: 5′-AGGTCGGTGTGAACGGATTTG-3′.

GAPDH-R: 5′-TGTAGACCATGTAGTTGAGGTCA-3′.

GAPDH was used as the internal reference gene. Relative gene expression was calculated using the 2^−ΔΔCt method.

### Animal experiments and tissue analysis

All animal experiments were approved by the Animal Ethics Committee of The First Affiliated Hospital of Bengbu Medical University (Approval No. 2024 − 286). C57BL/6 mice (male, 8 weeks old, weighing 22–25 g) were purchased from Vital River Laboratory (Beijing, China). Mice were housed under standard conditions with a 12/12-hour light/dark cycle and provided with food and water ad libitum. Mice were anesthetized using a mixture of 2% isoflurane and oxygen. A spinal cord contusion injury model was established at the T9-T10 level by applying a 200 kdyn force using an IH Impactor (Precision Systems & Instrumentation, Fairfax Station, VA, USA). Immediately after injury, mice in the designated groups received a local injection at the injury epicenter. The injection profile was as follows: a volume of 5 µL of the hydrogel precursor solution (10% w/v GelMA or Res@GelMA in PBS, pH 7.4) was delivered using a 30-gauge needle attached to a Hamilton microsyringe. The injection was performed at a slow, steady rate over 1 min, and the needle was held in place for an additional 1 min before withdrawal to prevent backflow. The hydrogel was then photo crosslinked in situ by exposing the surgical site to 365 nm UV light (10 mW/cm²) for 60 s. Postoperative care was rigorously provided. Manual bladder expression was performed twice daily until spontaneous voiding returned. Analgesia (buprenorphine, 0.1 mg/kg) was administered subcutaneously every 12 h for 3 days post-surgery. Animals were monitored daily for signs of infection or distress.

A total of forty mice were randomly assigned into five experimental groups (*n* = 8 per group) using a random number table: Sham operation (laminectomy only), Injury model (Model), Model + GelMA, Model + Res (5 mg/kg, intraperitoneal injection), and Model + Res@GelMA. The Basso Mouse Scale (BMS) locomotor rating was performed weekly by two investigators who were blinded to the group allocation of the animals.

After 4 weeks, mice were euthanized. Serum was collected for cytokine analysis. A consistent 5-mm segment of spinal cord tissue centered on the injury epicenter was dissected from each animal to ensure anatomical consistency. For histological analysis, the tissue was fixed in 4% paraformaldehyde, paraffin-embedded, and sectioned. For protein analysis, the tissue was snap-frozen in liquid nitrogen and stored at -80 °C until use.

### Statistical analysis

Data are shown as mean ± standard deviation (SD) for at least three independent experiments. The sample size (n) for each experiment is provided in the corresponding figure legend. All statistical analyses were performed using GraphPad Prism 9.0 software. The normality of data distribution was confirmed using the Shapiro-Wilk test. For comparisons between two groups, Student’s t-test (for parametric data) was used. For comparisons among more than two groups, one-way analysis of variance (ANOVA) was used, followed by Tukey’s post-hoc test for multiple comparisons. Comparisons between two groups were analyzed using Student’s t-test. A p-value < 0.05 was considered statistically significant (denoted as *p < 0.05, **p < 0.01, ***p < 0.001).

## Results

### Successful preparation and characterization of Res@GelMA

**Fig. 1:**
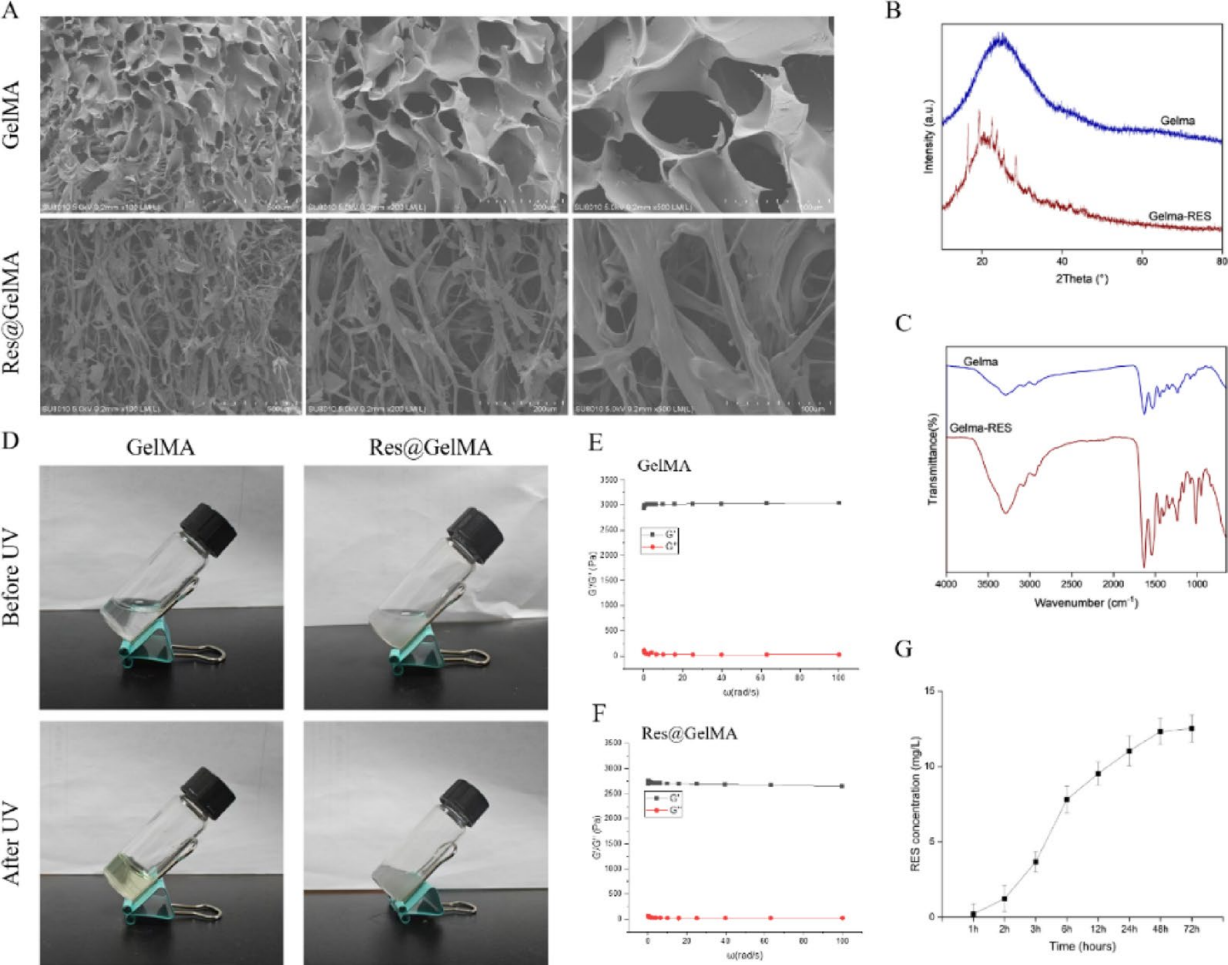
Successful preparation and characterization of Res@GelMA. **A** SEM images showing the porous microstructure of GelMA and Res@GelMA hydrogels. Res@GelMA exhibits a more compact structure with embedded Res particles. **​​B** XRD patterns reveal the crystal structures of GelMA and Res@GelMA. **​​C** FT-IR spectra confirming the successful incorporation of Res into GelMA through characteristic absorption bands. **​​D** Photographs of the UV-curing process, demonstrating the formation of Res@GelMA hydrogel under UV light. ​​**E**,** F** Dynamic rheological measurements showing the storage modulus (G’) and loss modulus (G’’) of GelMA (E) and Res@GelMA (F) during the UV-curing process, indicating the formation of a stable hydrogel network. **​​G** HPLC quantification of Res release from Res@GelMA over time, showing a sustained release profile. Data are representative of at least three independent experiments or preparations (*n* = 3). Data in (G) are presented as mean ± SD.*Abbreviations: Res (Resveratrol), GelMA (Gelatin methacryloyl), SEM (Scanning Electron Microscopy), XRD (X-ray diffraction), FT-IR (Fourier-transform infrared spectroscopy), UV (Ultraviolet), HPLC (High-Performance Liquid Chromatography)

To obtain a biomaterial with good therapeutic efficacy, we designed and prepared a Res-loaded methacrylated gelatin hydrogel (Res@GelMA). SEM results showed that both GelMA and Res@GelMA exhibited a well-defined porous network structure, and the addition of Res did not significantly alter their morphology (Fig. 1A). XRD patterns revealed the disappearance of characteristic peaks of crystalline Res, indicating successful encapsulation of Res within the GelMA matrix (Fig. 1B). FT-IR spectroscopy also indicated possible interactions, such as hydrogen bonding, between the components (Fig. 1C). Investigation of the photocuring properties clearly demonstrated the transition of the Res@GelMA hydrogel from a sol state to a solid gel upon UV light irradiation (Fig. 1D). Rheological experiments demonstrated that both GelMA and Res@GelMA displayed standard hydrogel rheological behavior, with their storage modulus (G’) consistently greater than their loss modulus (G’’), indicating good mechanical strength and elasticity (Fig. 1E, F). High-performance liquid chromatography (HPLC) analysis generated the in vitro release profile of Res from the GelMA hydrogel, demonstrating its sustained and slow release (Fig. 1G). Collectively, these data fully support the potential of the prepared hydrogel as an effective drug delivery system.

### Res@GelMA modulates macrophage inflammatory response and promotes M2 polarization

**Fig. 2:**
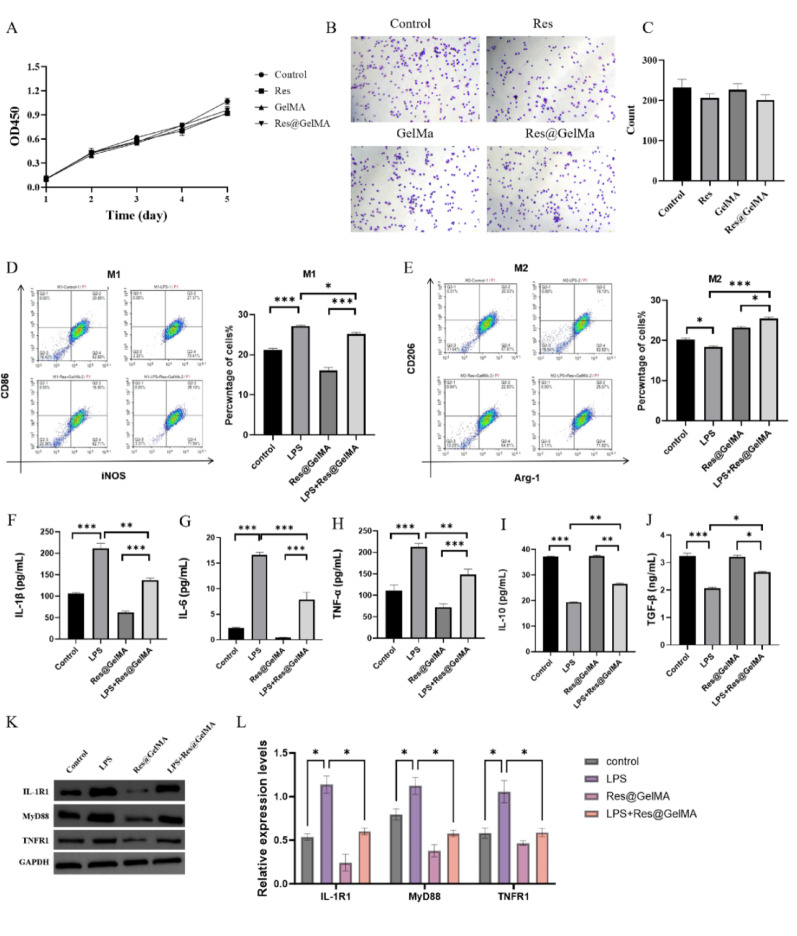
Res@GelMA modulates macrophage inflammatory response and promotes M2 polarization. **A** CCK8 assay showing that Res@GelMA does not affect RAW264.7 macrophage viability. **B**, **C** Cell adhesion assay demonstrating that Res@GelMA promotes macrophage adhesion. Representative images (**B**) and quantitative analysis **C** are shown. **D**, **E** Flow cytometry analysis of M1 (**D**) and M2 (E) polarization markers in RAW264.7 cells. Res@GelMA significantly promotes M2 polarization. **F**–**J** Clustered bar chart showing ELISA measurements of cytokine levels: pro-inflammatory cytokines IL-1β (**F**), IL-6 (**G**), TNF-α **H** and anti-inflammatory cytokines IL-10 **I**, TGF-β (**J**). Res@GelMA treatment reduces pro-inflammatory cytokine levels and enhances anti-inflammatory cytokine levels. **K**, **L** Western blot analysis of key inflammatory signaling proteins (IL-1R1, MyD88, TNFR1) and their grayscale quantification. Res@GelMA downregulates the expression of these proteins. GAPDH was used as an internal control. Data are from 3 independent experiments and are presented as mean ± SD. Statistical significance was determined by one-way ANOVA followed by Tukey’s post-hoc test (or Student’s t-test for comparisons between two groups). *P < 0.05, **P < 0.01, ***P < 0.001. CCK8 (Cell Counting Kit-8), ELISA (Enzyme-linked immunosorbent assay), IL-1β (Interleukin-1 beta), IL-6, (Interleukin-6); TNF-α, (Tumor necrosis factor-alpha); IL-10,(Interleukin-10); TGF-β, (Transforming growth factor-beta); LPS (Lipopolysaccharide); IL-1R1, (Interleukin-1 receptor type 1); MyD88, (Myeloid differentiation primary response 88); TNFR1, (Tumor necrosis factor receptor 1)

To further investigate the effects of Res@GelMA on RAW264.7 cells, we first assessed its potential impact on cell viability and adhesion capacity using the CCK-8 assay and cell adhesion assay. Results showed that Res@GelMA possessesed good cytocompatibility (Fig. 2A–C). Subsequently, flow cytometry was used to detect the expression of M1-associated surface markers (CD86, iNOS) and M2-associated markers (CD206, Arg-1). The results indicated that Res@GelMA effectively promoted M2 polarization (Fig. 2D and E). Furthermore, ELISA results showed that compared to the control group, Res@GelMA treatment reduced the expression of LPS-induced pro-inflammatory cytokines (IL-1β, IL-6, TNF-α) and increased the expression of anti-inflammatory cytokines (IL-10, TGF-β) (Fig. 2F–J). Western blot analysis further evaluated the effect of Res@GelMA on key inflammatory pathways, revealing that Res@GelMA significantly downregulated the expression of critical receptors and adaptor proteins closely associated with inflammation initiation, namely IL-1R1, MyD88, and TNFR1 (Fig. 2K-N). These results demonstrate that Res@GelMA exerts anti-inflammatory effects by influencing multiple key aspects of macrophage polarization and inflammation.

### Transcriptomic screening identifies Clec7a as a key DEG regulated by Res@GelMA

**Fig. 3:**
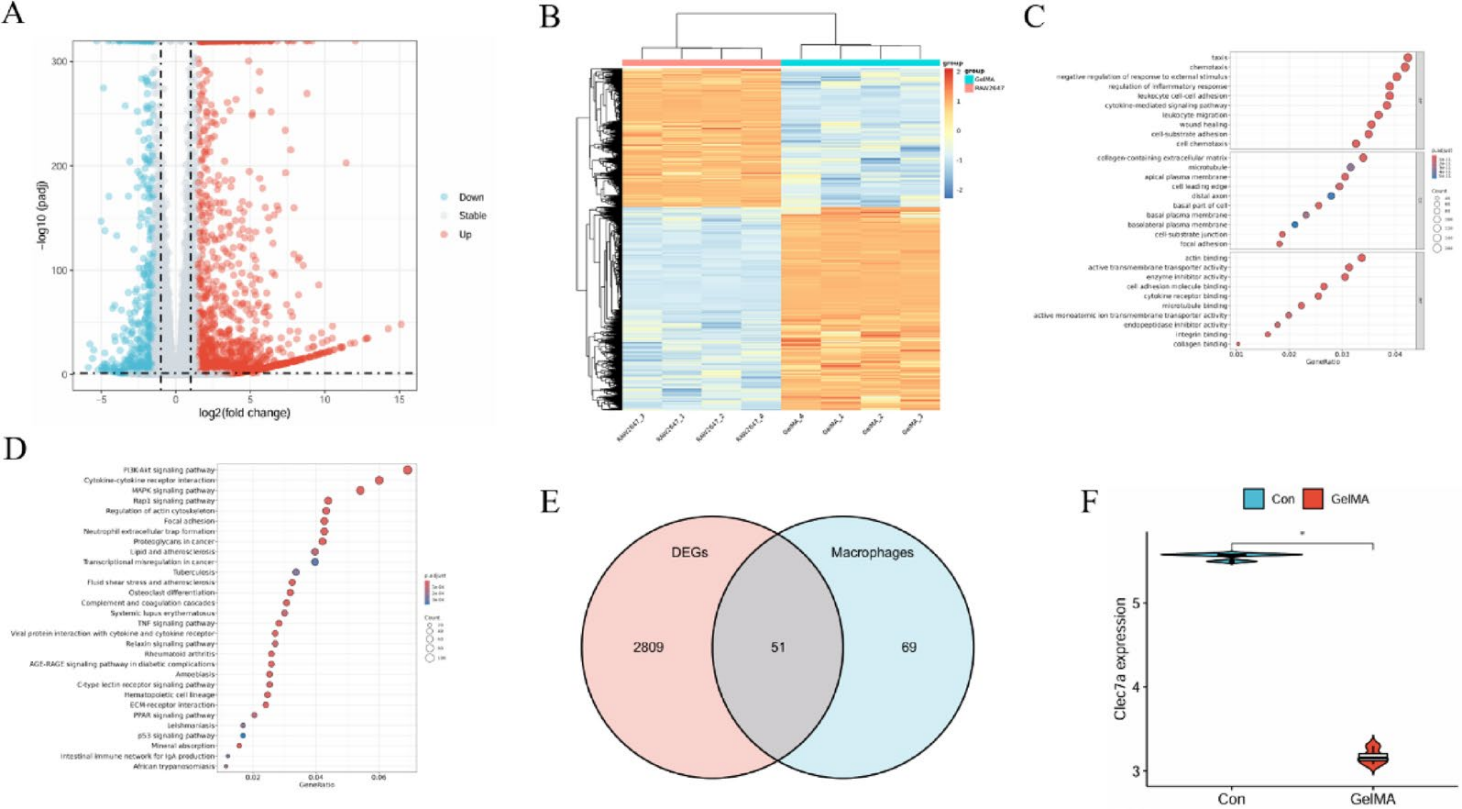
Transcriptomic screening identifies Clec7a as a Key DEG regulated by Res@GelMA. **A** Volcano plot of differentially expressed genes in mRNA sequencing (threshold: |log2FC| > 1 and *p* < 0.05, DESeq2 analysis). **B** Heatmap of DEGs, showing distinct gene expression patterns between control and Res@GelMA groups. **C** GO functional enrichment analysis. **D** KEGG pathway enrichment analysis. **E** Screening of differentially expressed genes related to macrophages. The RNA sequencing data were generated from three biologically independent samples per group. DEG, (Differentially expressed gene); Clec7a, (C-type lectin domain family 7 member A); GO, (Gene Ontology); KEGG, (Kyoto Encyclopedia of Genes and Genomes)

To delve deeper into the molecular mechanisms by which Res@GelMA regulates macrophage inflammatory responses, we performed mRNA transcriptome sequencing analysis on macrophages treated with either Control or Res@GelMA. The volcano plot revealed that Res@GelMA treatment caused significant differential expression in 2860 genes, with 1816 upregulated and 1044 downregulated. A heatmap clearly illustrated the clustering pattern of these differentially expressed genes (DEGs) (Fig. 3A and B). Gene Ontology (GO) functional enrichment analysis indicated that these DEGs were primarily enriched in biological processes such as chemotaxis, negative regulation of inflammatory response, and leukocyte cell-cell adhesion (Fig. 3C). Kyoto Encyclopedia of Genes and Genomes (KEGG) pathway enrichment analysis showed significant enrichment in pathways including the PI3K-Akt signaling pathway, cytokine-cytokine receptor interaction, MAPK signaling pathway, Rap1 signaling pathway, and regulation of the actin cytoskeleton (Fig. 3D). This suggests Res@GelMA has broad and profound effects on macrophage function. To focus on core targets highly relevant to macrophage function, we used a Venn diagram to identify 51 macrophage-associated DEGs and visualized their expression patterns using violin plots (Fig. 3E, Supplementary Figure S1, S2 and Supplementary Table 1). Among these, the Clec7a gene was selected as the core candidate target gene for subsequent research due to its significant downregulation by Res@GelMA treatment and its established association with inflammation and cell polarization in the literature (Fig. 3F).

### Clec7a plays a key role in the anti-inflammatory effects mediated by Res@GelMA

**Fig. 4:**
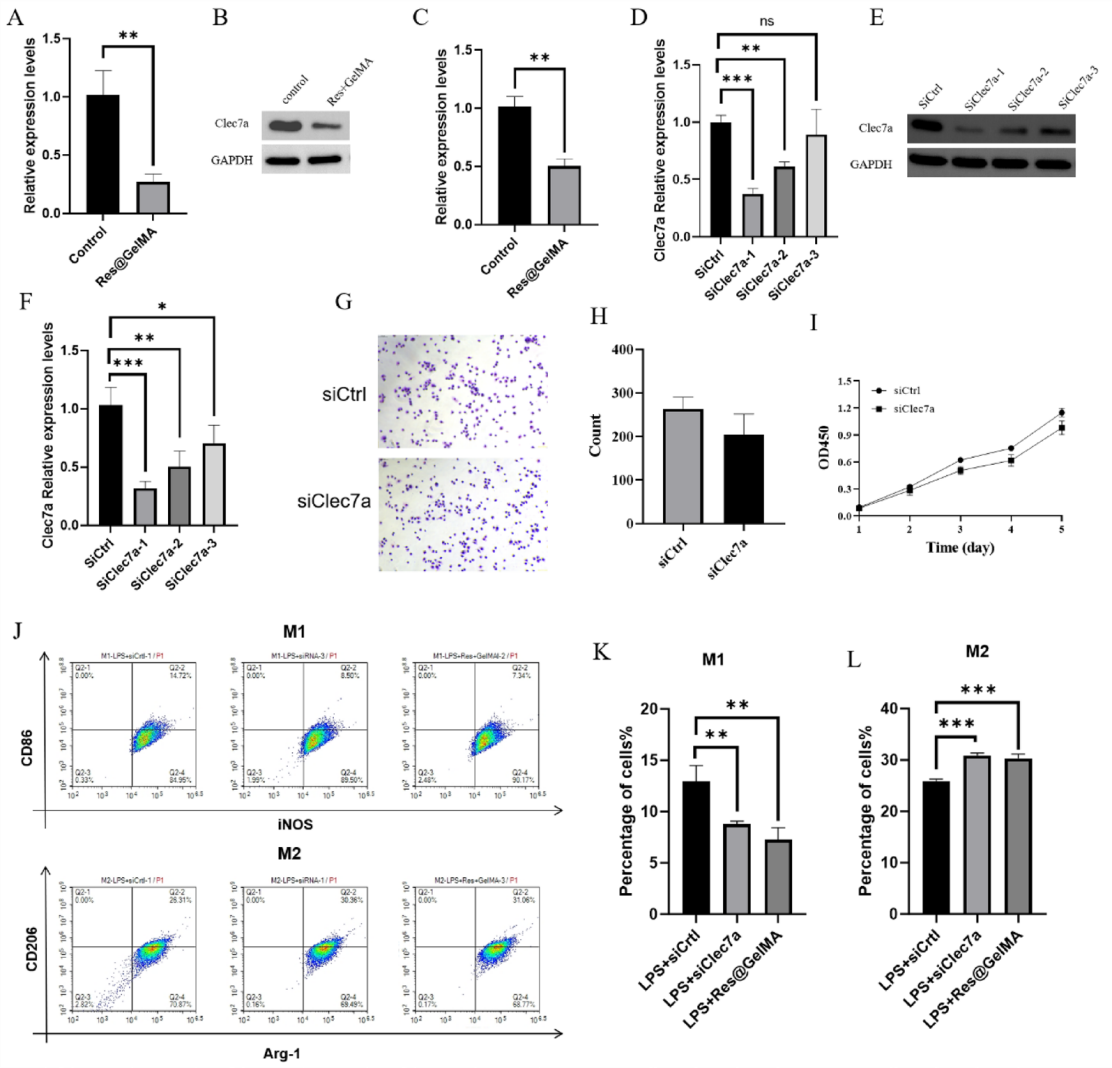
Clec7a plays a key role in the anti-inflammatory effects mediated by Res@GelMA. **A** qPCR detection of Clec7a expression in RAW264.7 cells after treatment with Control and Res@GelMA groups. **B** Western blot analysis of Clec7a expression in RAW264.7 cells after treatment with the control and Res@GelMA groups. **C** Grayscale analysis of B, with GAPDH as the internal control. **D** qPCR analysis of the effects of three siRNA knockdowns of Clec7a. **E** Western blot analysis of the effects of three siRNA knockdowns of Clec7a. **F** Grayscale analysis of (**E**), with GAPDH as the internal control. **G** Cell adhesion assay to examine the effects of siCtrl and siClec7a on RAW264.7 cell adhesion. **H** Statistical histogram of (**G**). **I** CCK8 assay to examine the effects of siCtrl and siClec7a on RAW264.7 cell viability. **J** Flow cytometry analysis of M1 and M2 polarization in RAW264.7 cells after treatment with LPS + siCtrl, LPS + siClec7a, and LPS + Res@GelMA. **K** Histogram of M1 polarization in J. **L** Histogram of M2 polarization in (**J**). Data are from 3 independent experiments and are presented as mean ± SD. Statistical significance was determined by one-way ANOVA followed by Tukey’s post-hoc test (or Student’s t-test for comparisons between two groups). *P < 0.05, **P < 0.01, ***P < 0.001. Clec7a, (C-type lectin domain family 7 member A); qPCR, (Quantitative polymerase chain reaction); siRNA, (Small interfering RNA)

qPCR and Western blot analysis further confirmed that Res@GelMA treatment significantly reduced the expression of Clec7a in macrophages at both the gene and protein levels (Fig. 4A–C). To elucidate the functional role of Clec7a, we designed small interfering RNAs (siRNAs) targeting Clec7a for knockdown experiments. Both qPCR and Western blot results validated the effective suppression of Clec7a expression by the siRNAs. SiClec7a-1 exhibited the best knockdown efficiency and was used in subsequent experiments (Fig. 4D–F). Functionally, Clec7a knockdown itself did not affect macrophage adhesion capacity or cell viability (Fig. 4G–I). Importantly, under LPS stimulation, siClec7a treatment significantly reduced macrophage polarization towards the pro-inflammatory M1 phenotype (CD86, iNOS) while promoting their shift towards the anti-inflammatory/reparative M2 phenotype (CD206, Arg-1) (Fig. 4J–L). This effect was highly consistent with that of direct Res@GelMA treatment, strongly suggesting that Clec7a downregulation is a core molecular event underlying Res@GelMA-mediated regulation of macrophage polarization.

### Res@GelMA regulates key inflammatory signaling pathways by targeting Clec7a

**Fig. 5:**
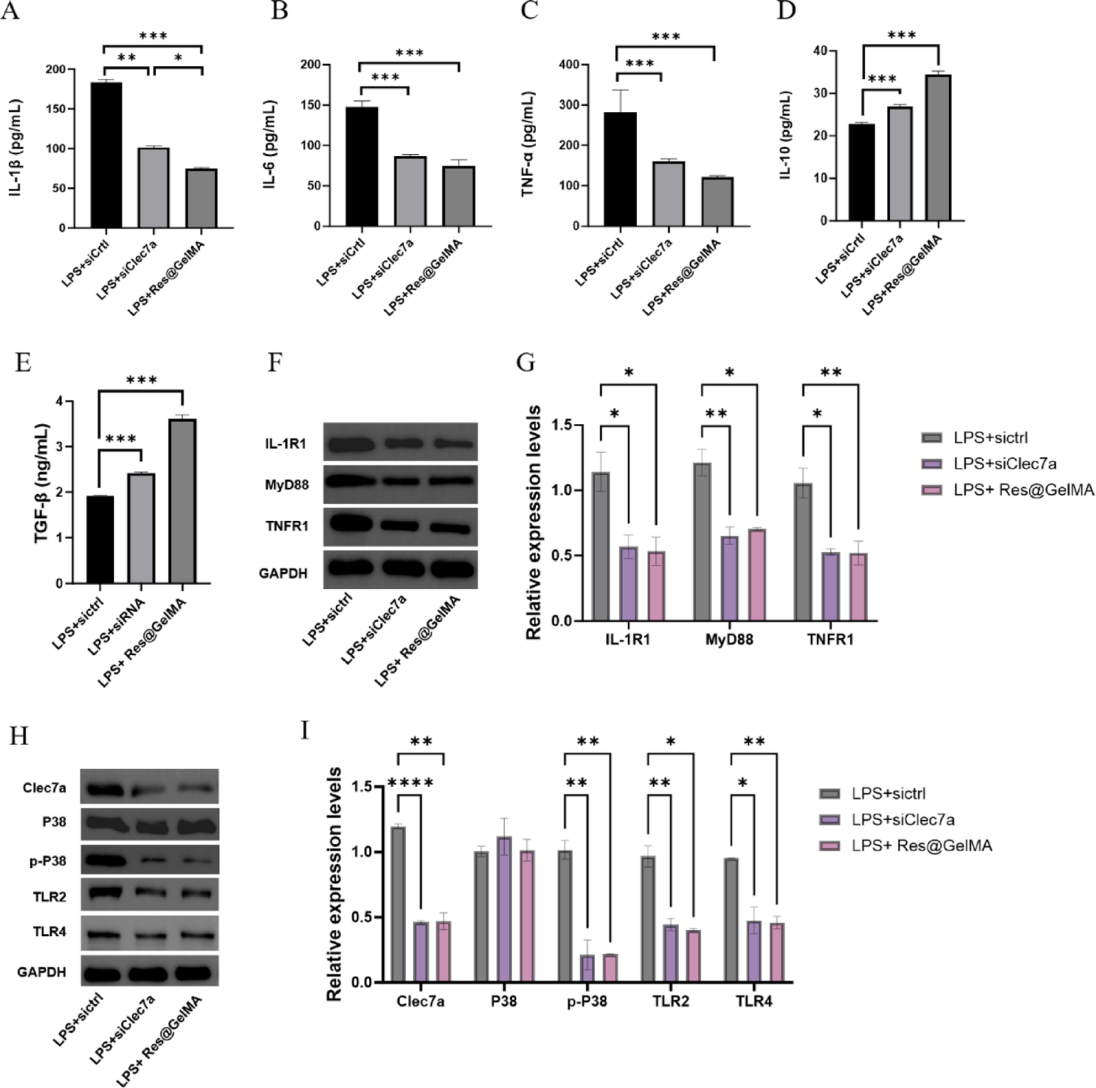
Res@GelMA regulates key inflammatory signaling pathways by targeting Clec7a.** A**–**E** Clustered bar chart of ELISA results showing cytokine levels in cell supernatants. Res@GelMA treatment and Clec7a knockdown similarly reduce pro-inflammatory cytokines (IL-1β, IL-6, TNF-α) and enhance anti-inflammatory cytokines (IL-10, TGF-β).** F**, **G** Western blot analysis and grayscale quantification of inflammatory signaling proteins (IL-1R1, MyD88, TNFR1). Both Res@GelMA treatment and Clec7a knockdown downregulate these proteins.** H**,** I** Western blot analysis and grayscale quantification of Clec7a and downstream signaling molecules (P38, p-P38, TLR2, TLR4). Res@GelMA treatment suppresses the Clec7a signaling pathway., GAPDH was used as the internal reference. Data are from 3 independent experiments and are presented as mean ± SD. Statistical significance was determined by one-way ANOVA followed by Tukey’s post-hoc test (or Student’s t-test for comparisons between two groups), *P < 0.05, **P < 0.01, ***P < 0.001. ELISA, (Enzyme-linked immunosorbent assay); IL-1R, (Interleukin-1 receptor type 1); MyD88, (Myeloid differentiation primary response 88); TNFR1, (Tumor necrosis factor receptor 1); TLR2, (Toll-like receptor 2); TLR4 (Toll-like receptor 4)

Having confirmed the function of Clec7a, we further dissected the downstream pathways regulated by Res@GelMA and Clec7a at the molecular mechanism level. ELISA results clearly demonstrated that Clec7a knockdown (LPS + siClec7a), consistent with Res@GelMA treatment, effectively inhibited the secretion of pro-inflammatory cytokines (IL-1β, IL-6, TNF-α) and promoted the release of anti-inflammatory cytokines (IL-10, TGF-β) (Fig. 5A-E). Western blot analysis with grayscale quantification further revealed that both Clec7a knockdown and Res@GelMA treatment significantly suppressed the expression levels of IL-1R1, MyD88, and TNFR1 proteins, indicating Clec7a acts as an upstream regulator of these key inflammatory receptors and adaptors (Fig. 5F-I). Furthermore, both Clec7a knockdown and Res@GelMA treatment significantly reduced the expression of Clec7a itself, as well as TLR2 and TLR4 (pattern recognition receptors potentially cooperating with Clec7a). Concurrently, both interventions markedly inhibited the phosphorylation level of the key pro-inflammatory kinase p38 MAPK (p-P38), without significantly affecting total P38 levels (Fig. 5J–O). These results suggest that Res@GelMA alleviates the inflammatory response by downregulating Clec7a, thereby suppressing TLR2/TLR4 expression and p38 MAPK phosphorylation.

### Res@GelMA demonstrates significant therapeutic efficacy in a mouse model of spinal cord injury

**Fig. 6:**
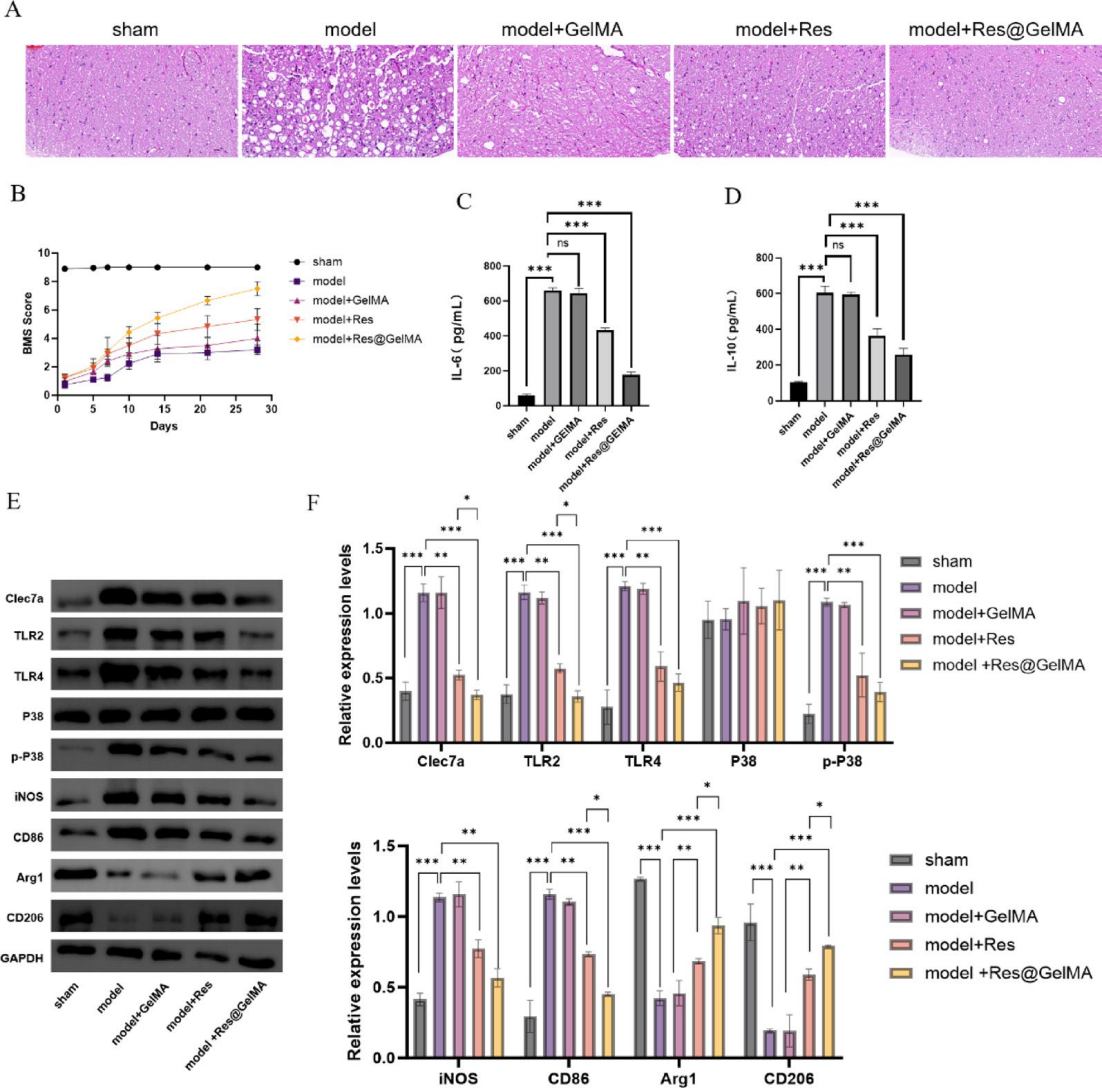
Res@GelMA demonstrates significant therapeutic efficacy in a mouse model of spinal cord injury. **A** HE staining of spinal cord tissues showing reduced tissue damage in the Res@GelMA treatment group. **B** Basso Mouse Scale (BMS) for hindlimb motor function. **C, D** ELISA was used to detect the levels of (**C**) IL-6 and (**D**) IL-10 in the serum of mice in sham, model, model + GelMA, model + Res, and model + Res@GelMA groups. **E**, **F** Western blot analysis and grayscale quantification of key proteins in spinal cord tissues. Res@GelMA treatment downregulates pro-inflammatory markers (Clec7a, TLR2, TLR4, p-P38, iNOS, CD86) and upregulates anti-inflammatory markers (Arg1, CD206). All in vivo assessments presented in this figure were performed at the endpoint of 28 days post-injury (dpi). Data are presented as mean ± SD (*n* = 8 mice per group). Statistical significance was determined by one-way ANOVA with Tukey’s post-hoc test., *P < 0.05, **P < 0.01, ***P < 0.001. HE, (Hematoxylin and eosin); BMS, (Basso Mouse Scale); IL-6, (Interleukin-6); IL-10, (Interleukin-10); iNOS, (Inducible nitric oxide synthase); CD86, (Cluster of differentiation 86); Arg1, (Arginase 1); CD206, (Macrophage mannose receptor 1)

Finally, we extended the application of the Res@GelMA hydrogel to an in vivo SCI model for therapeutic evaluation at 28 days post-injury. Hematoxylin and eosin (H&E) staining results showed that compared to the injury model group (Model), mice treated with Res@GelMA (Model + Res@GelMA) exhibited reduced inflammatory infiltration and significant amelioration of pathological damage in spinal cord tissue. This effect was superior to that of the GelMA alone (Model + GelMA) or free Res (Model + Res) treatment groups (Fig. 6A). BMS score, a crucial indicator of functional recovery, further confirmed that mice in the Res@GelMA treatment group showed significantly better recovery of hindlimb motor function compared to other treatment groups and the untreated model group (Fig. 6B). Serological ELISA detection revealed that the Model + Res@GelMA group had significantly lower serum levels of the pro-inflammatory cytokine IL-6 and significantly higher levels of the anti-inflammatory cytokine IL-10, indicating a beneficial systemic anti-inflammatory effect of the material (Fig. 6C, D). The most critical molecular mechanism validation came from Western blot analysis of the injury site tissue: results showed that Model + Res@GelMA treatment significantly downregulated the expression of Clec7a, TLR2, and TLR4, as well as the level of p-P38 in the injured spinal cord tissue. It also effectively suppressed the expression of pro-inflammatory M1 markers (iNOS, CD86) while significantly upregulating reparative M2 markers (Arg1, CD206), consistent with the in vitro results (Fig. 6F-N).

## Discussion

Although the pathological mechanism and treatment methods of SCI have been studied for decades, clinical translation is still slow [29–32]. The various methods currently used in clinical practice can only temporarily alleviate the patient’s condition or slow down the deterioration of the disease, but cannot effectively block the secondary injury cascade or promote the regeneration of central nervous system (CNS) neurons [7, 9]. Uncontrolled inflammatory response and imbalance of macrophage phenotype polarization are considered to be key drivers of secondary injury [33, 34]. A large number of experiments have shown that excessive expansion of M1 macrophages dominated by IL-6/IL-8/TNF-a/IFN-g in the early stage after injury can cause the death of neurons and oligodendrocytes and inhibit axonal remyelination [15]. However, due to the lack of targeting, systemic toxicity and short half-life, traditional anti-inflammatory drugs are difficult to maintain a stable effective concentration for a long time to achieve effective treatment. In recent years, targeted drug delivery technology based on biomaterials has opened up a new direction for us to solve this problem. Many scholars have done a lot of useful work in this regard [35]. However, their work only focuses on how to introduce exogenous anti-inflammatory factors into the body to play a role, but does not consider how to simultaneously regulate the inflammatory mediators secreted by macrophages themselves and their polarization state [36].

In this context, this study constructed a smart hydrogel Res@GelMA loaded with natural polyphenol Res. Different from traditional material systems that are simply used as drug carriers [37, 38], Res@GelMA has three advantages: (1) The photocurable GelMA scaffold mimics the extracellular matrix structure of the spinal cord, modulating the phenotype of macrophages that are recruited to the injury site [27, 39]; (2) The continuous release of Res can avoid the concentration fluctuations caused by the rapid metabolism of free drugs, and achieve long-term immune regulation; (3) Res itself has the potential for multi-target regulation, but previous studies have not clarified its specific molecular targets for macrophages [40]. Our findings on the efficacy of Res@GelMA are consistent with, yet significantly advance, previous efforts to harness resveratrol for SCI therapy. For instance, Zhao et al. demonstrated that free resveratrol inhibits inflammation after SCI via the SIRT-1/NF-κB pathway [25]. However, other delivery approaches, such as polymeric nanoparticles or liposomes, while improving solubility to some extent, often lack the capacity for localized, sustained release and the provision of a supportive 3D matrix for cell infiltration and tissue repair [41]. Our GelMA-based system uniquely combines the proven anti-inflammatory effects of resveratrol [25] with the benefits of an injectable, biomimetic hydrogel, thereby addressing the critical pharmacokinetic limitations of the free drug and offering a multifaceted therapeutic strategy that surpasses simple drug delivery. By integrating materials science and immunology analysis, we found that Res@GelMA has a significantly better therapeutic effect than the GelMA vector alone or free Res treatment group. It can significantly inhibit the polarization of macrophages to the pro-inflammatory M1 phenotype and enhance the polarization trend of RAW264.7 to the repair type M2. It is worth noting that its anti-inflammatory efficacy is comparable to the gene modification strategies reported in the literature [42], but avoids the safety risks and operational complexity of cell therapy.

At the mechanistic level, this study established the C-type lectin receptor Clec7a as the core target of Res@GelMA in regulating macrophage polarization through transcriptome sequencing. Clec7a, as a transmembrane protein, is expressed by macrophages and some other immune cells and is believed to control the innate immune response to pathogens and phagocytic properties [43]. Functional validation confirmed that Res@GelMA treatment can specifically downregulate Clec7a expression, which is consistent with a large number of studies reporting that knocking down Clec7a has the same anti-inflammatory effect as Res@GelMA treatment [44, 45]. We found that knocking down Clec7a significantly inhibited TLR2/4 expression and p38 phosphorylation, and downregulated inflammatory adaptor proteins such as MyD88, IL-1R1, and TNFR1, reducing IL-6/TNF-α release and promoting IL-10 secretion. Our study also showed that knocking down Clec7a did not affect the viability and adhesion ability of macrophages. However, this study has not yet clarified whether Clec7a downregulation affects classic downstream pathways such as Syk-CARD9. This not only reinforces Clec7a’s role as a central regulator of innate immunity but also validates it as a compelling target for immunomodulatory therapies in neural repair.

In the spinal cord contusion model, Res@GelMA showed significant advantages over the free Res group in terms of animal efficacy. The BMS score of the Res@GelMA local implantation group was significantly improved, the serum IL-6 level decreased, and the IL-10 level increased. In the present study, local implantation of Res@GelMA significantly improved the BMS score to 7.5 ± 0.41 at 28 dpi, compared to 3.2 ± 0.28 in the injury model group. This degree of functional recovery is comparable to, if not superior to, that achieved by other advanced strategies reported in the literature, such as the transplantation of bone marrow mesenchymal stem cell-derived exosomes (final BMS score ~ 5.8) [46]. This therapeutic advantage stems from the local sustained-release properties of the hydrogel, which makes up for the poor targeting of free Res and low oral bioavailability [26]. It is important to acknowledge a limitation of this study. While our data strongly suggest that functional recovery and immune regulation may play a neuroprotective role, we did not include direct histological evidence of neuronal or oligodendrocyte preservation, such as neuronal cell counts (e.g., NeuN staining), apoptosis detection (e.g., TUNEL assay), or quantitative analysis of axonal integrity (e.g., NF200) and myelination (e.g., MBP). Second, the functional improvement, while significant, was assessed behaviorally; complementary electrophysiological assessments would be required to confirm the restoration of neural conduction across the injury site. Since the primary focus of our study was on elucidating molecular and signaling mechanisms, the mouse model was chosen. Moreover, because functional recovery in mice is inherently less comprehensive than in rat models, future studies should also incorporate locomotor and electrophysiological assessments in rats and even larger animals to strengthen translational relevance. Future studies incorporating these direct morphological assessments would help supplement our findings and provide a more comprehensive understanding of the neuroprotective efficacy of Res@GelMA. Furthermore, this study focused only on a single chronic time point (4 weeks post-injury), failing to capture the dynamic changes in microglia/macrophage responses during the acute and subacute phases. Future studies should conduct detailed time-varying immunohistochemical analyses to elucidate the precise temporal effects of Res@GelMA on polarization kinetics and glial scar formation.

In summary, Res@GelMA breaks the previous situation of spinal cord injury being difficult to cure through a new perspective, namely, targeting macrophage polarization, inflammation and the Clec7a/TLR/p38 axis, which is of great significance. Although there are still many problems to be solved from mechanism analysis to clinical application, this work combines the three directions of material design, immune metabolic regulation and neural repair to achieve mutual promotion among the three, which will also provide certain guidance for our next step in developing a new generation of precise spinal cord injury treatment methods.

## Supplementary Information

Below is the link to the electronic supplementary material.

Supplementary Material 1

Supplementary Material 2

Supplementary Material 3

## Data Availability

All other relevant data are available from the corresponding author upon reasonable request.
