# Supplementary material for: Clec7a-targeted Res@GelMA hydrogels regulate macrophage polarization to reduce neuroinflammation and promote spinal cord repair
Source: J Orthop Surg Res. 2026 Jan 24;21:133. doi: 10.1186/s13018-025-06631-0 (PMC12911380; doi:10.1186/s13018-025-06631-0)
Supplement: Supplementary file 1 — Supplementary Material 1 [file 13018_2025_6631_MOESM1_ESM.docx]

**Supplementary Materials:**


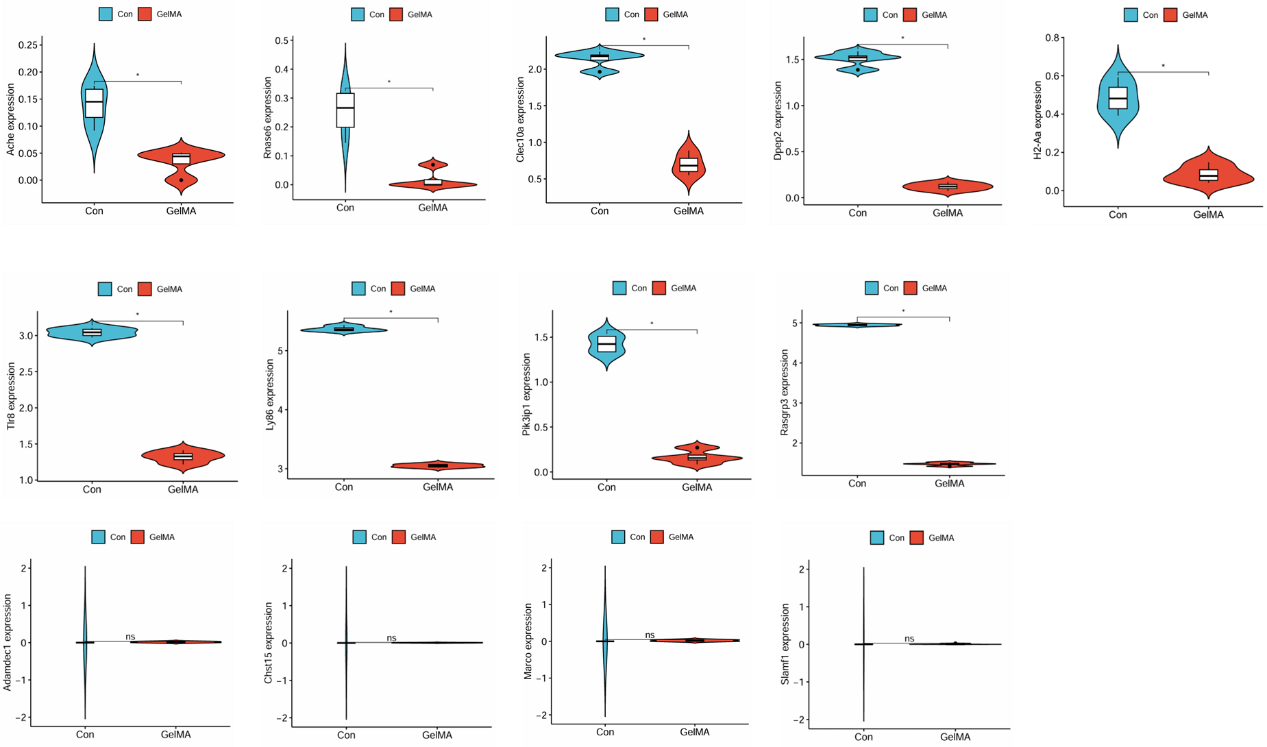


**Figure S1. Violin plot of macrophage-related DEG expression (10 were downregulated and 4 had no significant differences).**


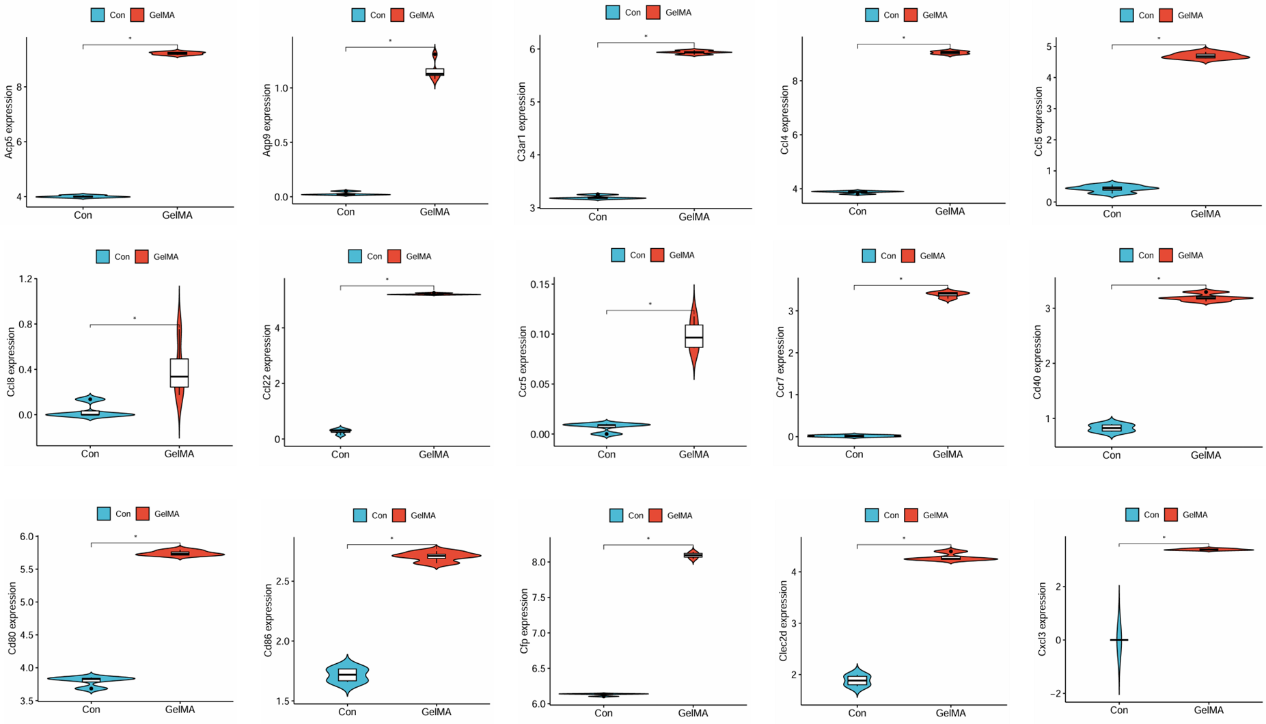


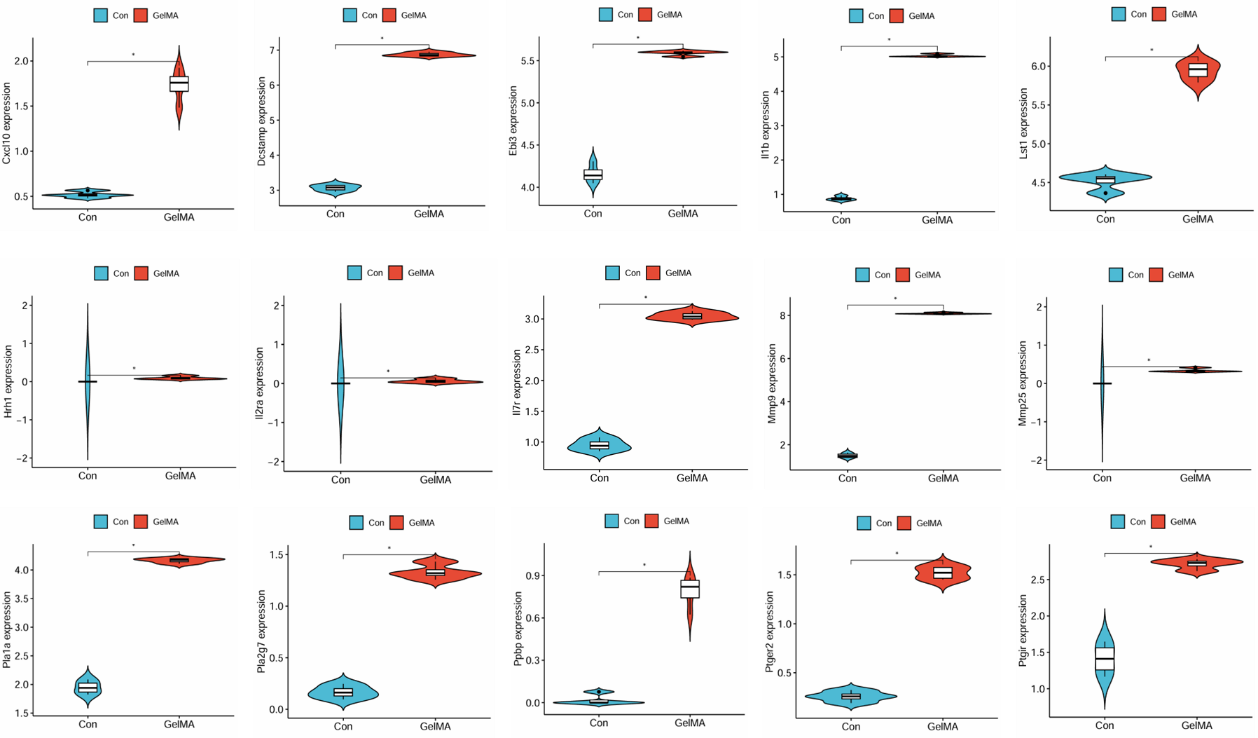


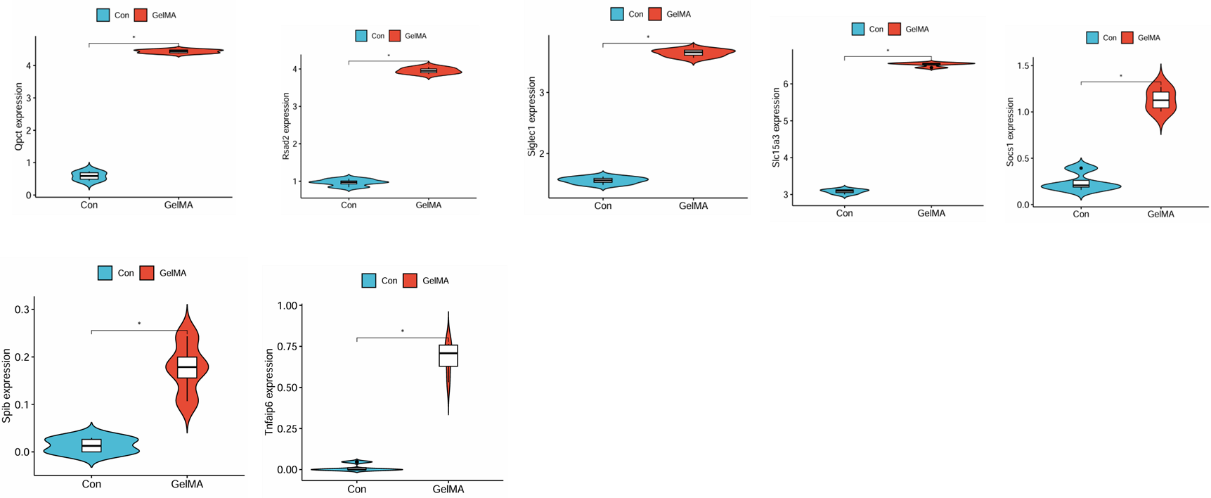


**Figure S2. Violin plots of 37 upregulated macrophage-related DEGs.**
