# Supplementary figures and images for: Clec7a-targeted Res@GelMA hydrogels regulate macrophage polarization to reduce neuroinflammation and promote spinal cord repair
Source: J Orthop Surg Res. 2026 Jan 24;21:133. doi: 10.1186/s13018-025-06631-0 (PMC12911380; doi:10.1186/s13018-025-06631-0)

Figure 2K:


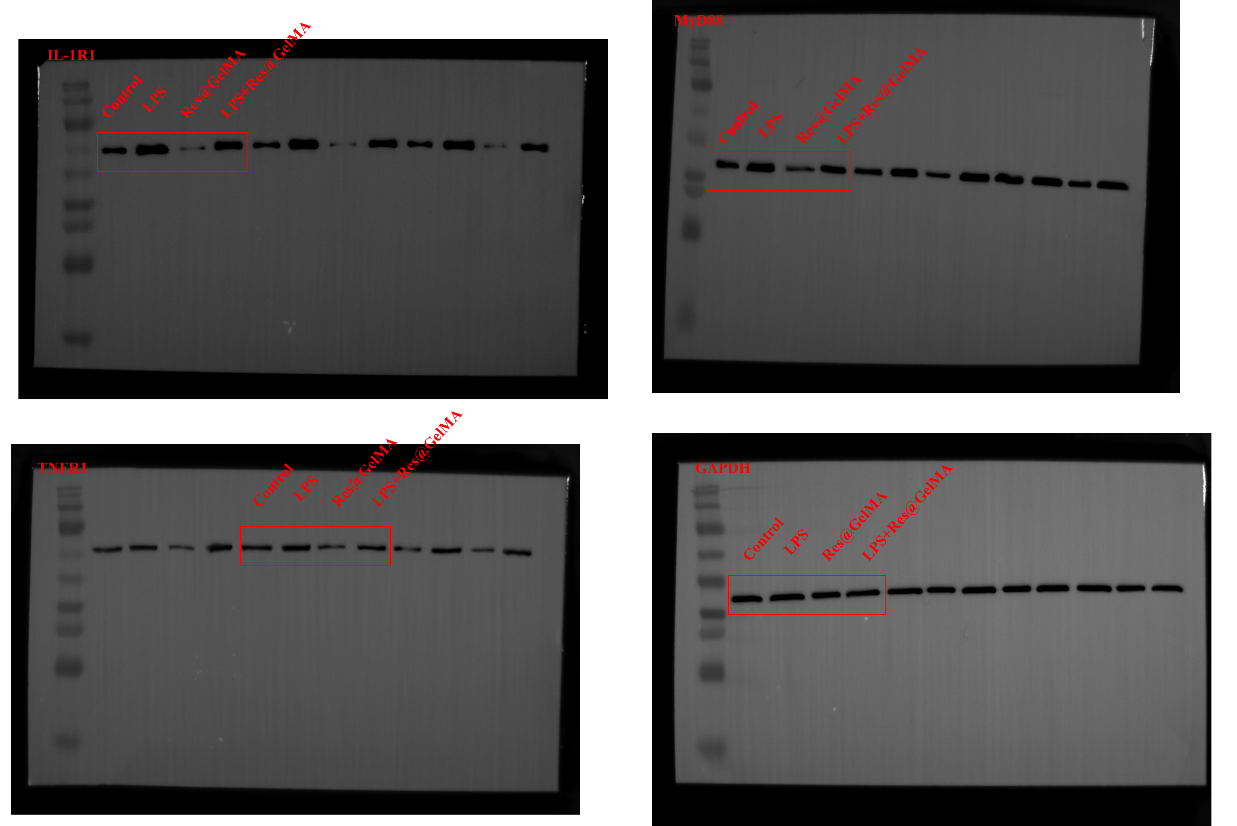


Figure 4B：


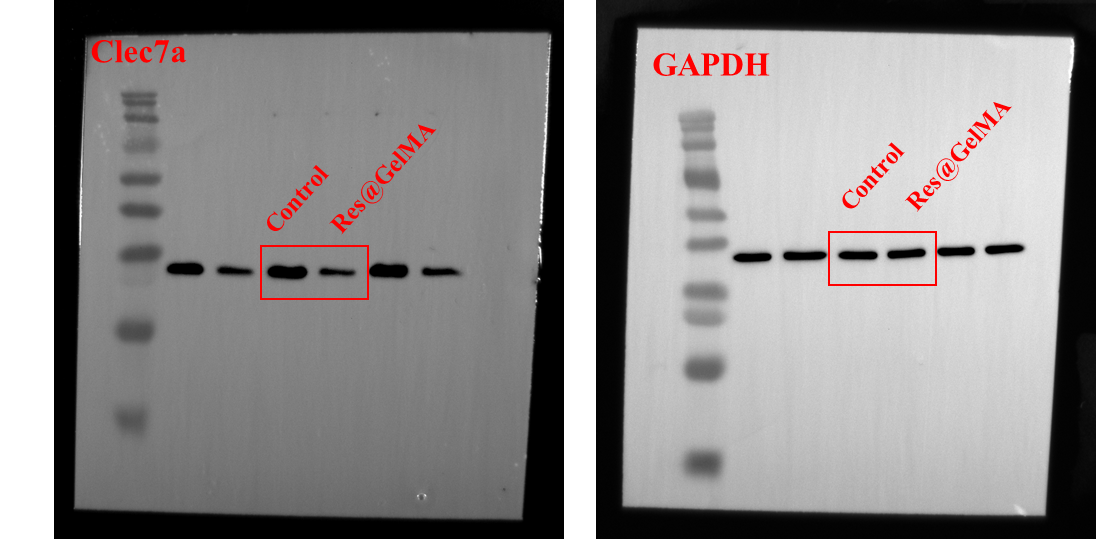


Figure 4E：


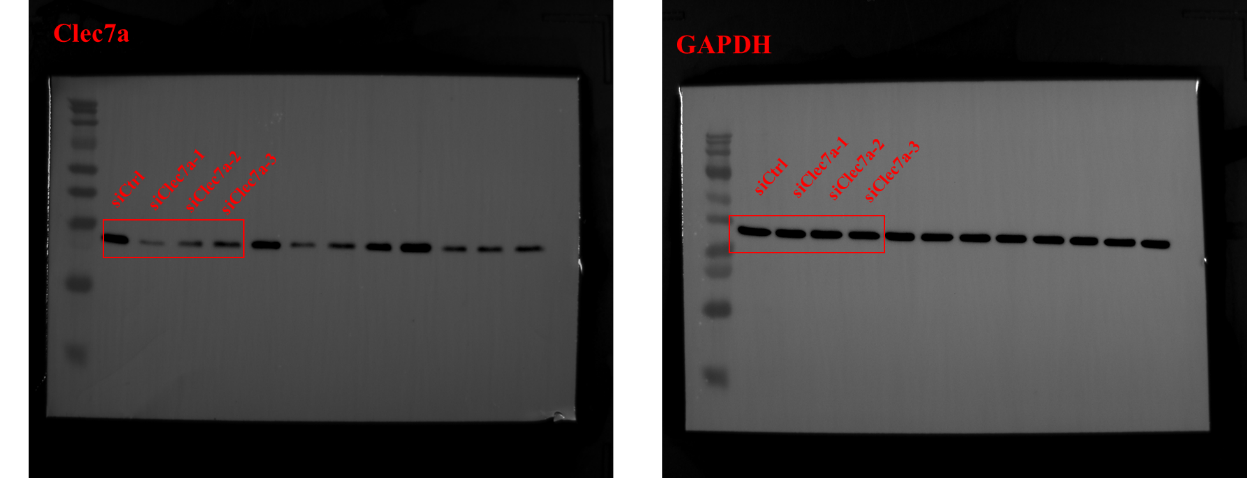


Figure 5F:


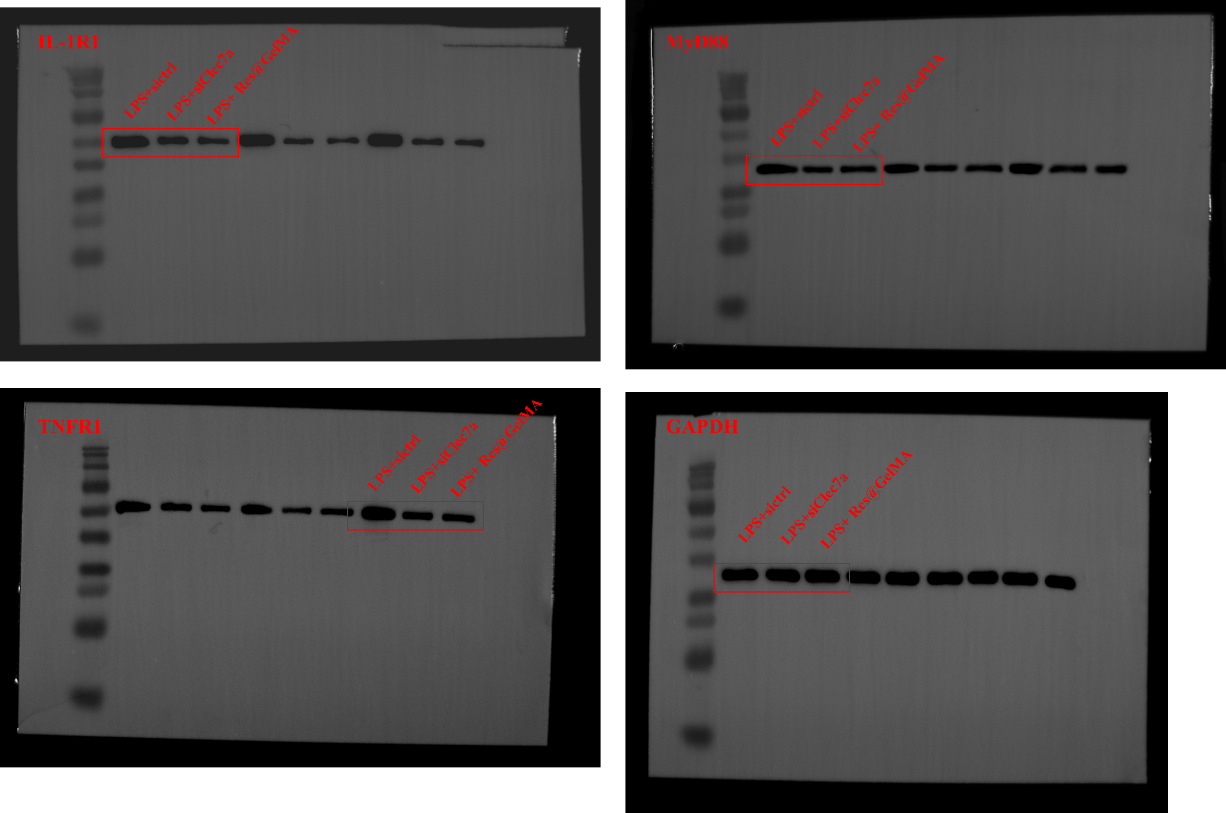


Figure 5J:


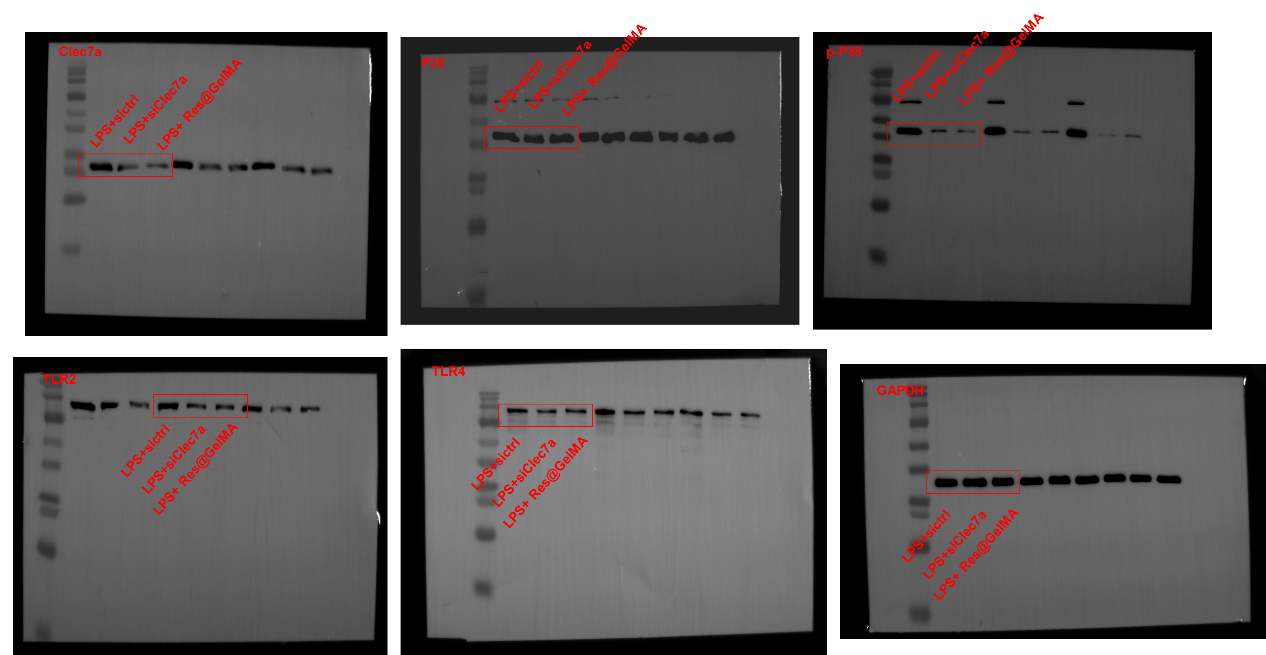


Figure 6E:


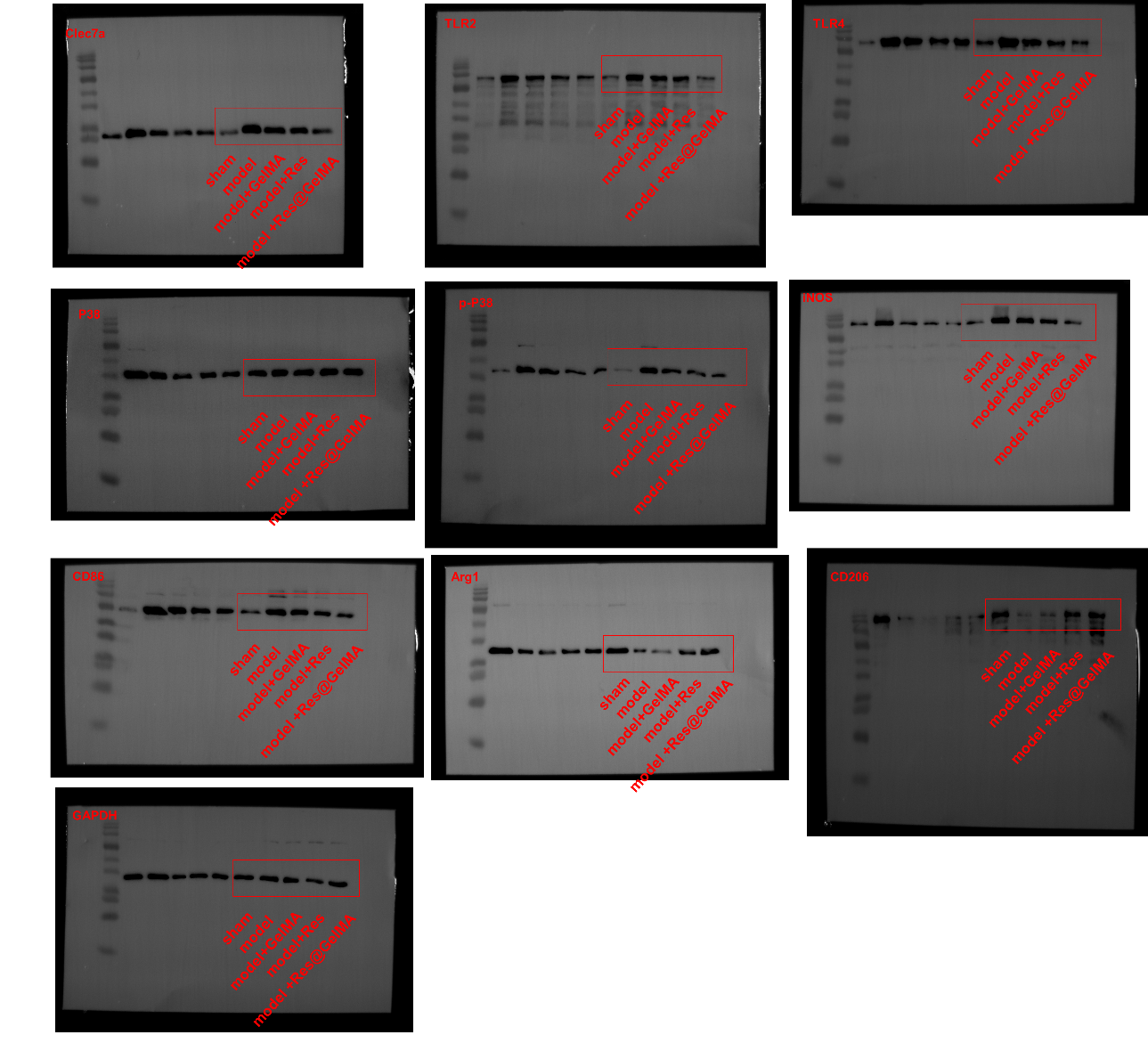

Supplement: Supplementary file 2 — Supplementary Material 2 [file 13018_2025_6631_MOESM2_ESM.docx]
