# Supplementary material for: Clec7a-targeted Res@GelMA hydrogels regulate macrophage polarization to reduce neuroinflammation and promote spinal cord repair
Source: J Orthop Surg Res. 2026 Jan 24;21:133. doi: 10.1186/s13018-025-06631-0 (PMC12911380; doi:10.1186/s13018-025-06631-0)
Supplement: Supplementary file 3 — Supplementary Material 3 [file 13018_2025_6631_MOESM3_ESM.docx]

Supplementary Table 1. 51 macrophage-related DEGs.

| **Gene** | **Log2 Fold Change** | **p-value** | **change** |
| --- | --- | --- | --- |
| Clec10a | -2.15 | ＜0.001 | Down |
| Cfp | 2.28 | ＜0.001 | Up |
| Acp5 | 5.61 | ＜0.001 | Up |
| Pla1a | 2.86 | ＜0.001 | Up |
| Ebi3 | 1.78 | ＜0.001 | Up |
| Il7r | 3.26 | ＜0.001 | Up |
| Spib | 4.07 | ＜0.001 | Up |
| Ccl8 | 3.67 | ＜0.001 | Up |
| Slamf1 | 1.8 | ＜0.001 | Up |
| Cd40 | 3.69 | ＜0.001 | Up |
| Mmp9 | 7.51 | ＜0.001 | Up |
| Ccl4 | 5.56 | ＜0.001 | Up |
| Rsad2 | 4.22 | ＜0.001 | Up |
| Ly86 | -2.17 | ＜0.001 | Down |
| Rnase6 | -3.84 | ＜0.001 | Down |
| Adamdec1 | 1.78 | ＜0.001 | Up |
| Dcstamp | 4.28 | ＜0.001 | Up |
| Cd86 | 1.57 | ＜0.001 | Up |
| Ache | -1.74 | ＜0.001 | Down |
| Mmp25 | 7.42 | ＜0.001 | Up |
| Pla2g7 | 3.93 | ＜0.001 | Up |
| Qpct | 5.63 | ＜0.001 | Up |
| Slc15a3 | 3.9 | ＜0.001 | Up |
| Marco | 1.82 | ＜0.001 | Up |
| Il2ra | 4.94 | ＜0.001 | Up |
| Siglec1 | 2.86 | ＜0.001 | Up |
| Il1b | 5.53 | ＜0.001 | Up |
| Ppbp | 5.86 | ＜0.001 | Up |
| Cxcl3 | 11.98 | ＜0.001 | Up |
| Clec2d | 3.08 | ＜0.001 | Up |
| Chst15 | 1.82 | ＜0.001 | Up |
| Ccl22 | 7.71 | ＜0.001 | Up |
| Aqp9 | 6.27 | ＜0.001 | Up |
| Pik3ip1 | -3.43 | ＜0.001 | Down |
| Cxcl10 | 2.73 | ＜0.001 | Up |
| Ccl5 | 6.49 | ＜0.001 | Up |
| H2-Aa | -2.43 | ＜0.001 | Down |
| Ptger2 | 3.55 | ＜0.001 | Up |
| Ccr7 | 9.71 | ＜0.001 | Up |
| Socs1 | 2.97 | ＜0.001 | Up |
| Tlr8 | -1.98 | ＜0.001 | Down |
| C3ar1 | 3.18 | ＜0.001 | Up |
| Ptgir | 2.03 | ＜0.001 | Up |
| Hrh1 | 5.23 | ＜0.001 | Up |
| Tnfaip6 | 6.25 | ＜0.001 | Up |
| Dpep2 | -4.1 | ＜0.001 | Down |
| Rasgrp3 | -3.77 | ＜0.001 | Down |
| Lst1 | 1.76 | ＜0.001 | Up |
| Cd80 | 2.32 | ＜0.001 | Up |
| Ccr5 | 4.17 | ＜0.001 | Up |
| Clec7a | -2.22 | ＜0.001 | Down |
